# Effect of wavelength and liquid on formation of Ag, Au, Ag/Au nanoparticles via picosecond laser ablation and SERS-based detection of DMMP

**DOI:** 10.3762/bjnano.15.86

**Published:** 2024-08-19

**Authors:** Sree Satya Bharati Moram, Chandu Byram, Venugopal Rao Soma

**Affiliations:** 1 Advanced Centre for Research in High Energy Materials (ACRHEM), DRDO Industry Academia - Centre of Excellence (DIA-COE), University of Hyderabad, Prof. C. R. Rao Road, Hyderabad 500046, Telangana, India; 2 Department of Physics, Indian Institute of Technology Hyderabad, Kandi 502285, Telangana, India; 3 Department of Physics, College of Arts and Sciences, University of Dayton, 300 College Park, Dayton, Ohio 45469, USA; 4 School of Physics, University of Hyderabad, Prof. C. R. Rao Road, Hyderabad 500046, Telangana, India

**Keywords:** dimethyl methyl phosphonate, laser material interaction, metal nanoparticles, picosecond laser ablation, SERS, thiram

## Abstract

The present study investigates the effects of input wavelength (1064, 532, and 355 nm) and surrounding liquid environment (distilled water and aqueous NaCl solution) on the picosecond laser ablation on silver (Ag), gold (Au), and Ag/Au alloy targets. The efficacy of the laser ablation technique was meticulously evaluated by analyzing the ablation rates, surface plasmon resonance peak positions, and particle size distributions of the obtained colloids. The nanoparticles (NPs) were characterized using the techniques of UV–visible absorption, transmission electron microscopy, and energy-dispersive X-ray spectroscopy. Furthermore, NPs of various sizes ranging from 6 to 35 nm were loaded onto a filter paper by a simple and effective drop-casting approach to achieve flexible surface-enhanced Raman spectroscopy (SERS) substrates/sensors. These substrates were tested using a simple, portable Raman device to identify various hazardous chemicals (malachite green, methyl salicylate, and thiram). The stability of the substrates was also systematically investigated by determining the decay percentages in the SERS signals over 60 days. The optimized SERS substrate was subsequently employed to detect chemical warfare agent (CWA) simulants such as methyl salicylate (a CWA simulant for sulfur mustard) and dimethyl methyl phosphonate (has some structural similarities to the G-series nerve agents) at different laser excitations (325, 532, and 633 nm). A notably higher SERS efficiency for CWA simulants was observed at a 325 nm Raman excitation. Our findings reveal that a higher ablation yield was observed at IR irradiation than those obtained at the other wavelengths. A size decrease of the NPs was noticed by changing the liquid environment to an electrolyte. These findings have significant implications for developing more efficient and stable SERS substrates for chemical detection applications.

## Introduction

Metal nanoparticles (NPs) are versatile materials widely used across various scientific and technological fields due to their distinctive optical, physical, and chemical properties. Over the past few decades, different methods have been developed for NP synthesis, including chemical reduction, electrochemistry, atomic layer deposition, laser ablation synthesis in solution (LASiS), and sputtering [1]. The LASiS technique has been proven to be cost-effective in producing various shapes of NPs with distinct size distributions in a short time (a few minutes). It offers many advantages including high purity, minimal contamination, and precise control over NP size and composition, making it a preferred choice for nanomaterials synthesis [2–5]. The process involves laser plasma interacting with a metal in a liquid; it excites electrons, which then generates atomic vibrations within a few picoseconds, causing rapid heating, melting, and explosive decomposition of the metal surface. This results in an explosive ejection of vapor and liquid from the surface. The metal plume cannot freely expand in water and is slowed down, forming a hot metal layer at the water interface. The hot metal layer heats the water to a supercritical state, mixing metal atoms with water. The expanding metal/water mixture promotes rapid nucleation and growth of small metal NPs and contributes to forming a cavitation bubble. The hot metal layer also breaks into larger droplets due to instabilities, creating NPs of different sizes within a few nanoseconds of laser exposure [6]. The properties of NPs, such as size, shape, crystallinity, productivity, and composition, can be influenced by several experimental parameters during synthesis [4,6–10]. The impact of laser parameters, such as pulse duration, wavelength, repetition rate, and fluence, and of the liquid parameter on NP productivity, shape, and size distribution remains an area of ongoing research [11–15]. Pulsed laser irradiation of liquids (PLIL) can affect the size and shape of NPs. Various approaches are described in the literature, such as (i) laser fragmentation in liquid (LFL), (ii) laser melting in liquid (LML), and (iii) laser defect engineering in liquid (LDL) [16]. In our previous work, we fabricated Ag–Cu alloy NPs using the femtosecond (fs) laser irradiation approach [17]. Similarly, Ag/Au alloy NPs were fabricated by laser ablation of single metal targets in water followed by re-irradiation of mixed colloidal suspensions, as demonstrated by Compagnini et al. [18]. Additionally, Zhang et al. [19] reported the LML approach to synthesize germanium submicron spheres from picosecond (ps) laser irradiation of Ge powders containing nanoscale and microscale particles. Maximova et al. [20] achieved size-controllable Au NPs in stable solutions via femtosecond laser fragmentation, tuning sizes by adjusting fluence. This technique is employed to create various categories of alloy NPs [21]. Alloying by LASiS can mitigate undesired features associated with plasmonic materials, such as high cost, chemical instability, and sustainability issues [22–23]. Menéndez-Manjón et al. [24] reported the synthesis of Ag/Au alloy NPs via picosecond LASiS of solid targets in monomer MMA. Amendola et al. [25] demonstrated the fabrication of magneto-plasmonic alloy NPs, such as Fe–Au, by laser ablating Au/Fe multilayers with varying thicknesses and deposition orders. Jakobi et al. [26] reported the synthesis of Pt–Ir alloy NPs by femtosecond laser ablation of a Pt_9_Ir target in acetone and further utilized as PtIr electrodes. In recent years, laser-ablated NPs/NSs have gained prominent interest in many applications, such as photoelectronic devices, biochemical sensors, and surface-enhanced Raman spectroscopy (SERS) substrates, due to their high purity NPs as well as an easy method for altering the structures, NPs/NSs sizes, and morphology by tuning the laser parameters and surrounding media [27–29]. The SERS substrate efficiency mainly depends on the material, size, and shape of the NPs. Recent terrorist activities involving explosives and chemical warfare agents highlight the urgent need for sensitive and selective chemical sensors. These sensors must be using low power and be capable of trace detection. Dimethyl methyl phosphonate (DMMP) is commonly used as a less toxic simulant for sarin, a G-series nerve agent. DMMP can, in general, be used in making chemical weapons. Zheng et al. [30] reviewed various methods for DMMP detection, including mass-sensitive sensors, surface acoustic wave (SAW) sensors, microelectromechanical systems (MEMS), carbon nanotubes, and chemiresistive sensors. SERS-active substrates encounter obstacles in translation toward practical applications, primarily due to the difficulty of sample collection. As a result, there is a persistent need for affordable and accessible fabrication methods which guarantee stability and reproducibility along with accessible sample collection of SERS substrates. There has been significant interest in utilizing flexible materials such as paper, nitrocellulose, polymer film, cotton fabrics, adhesive tape, glass fibers, and biomaterials for constructing flexible SERS substrates, owing to their numerous advantages over traditional options such as glass and silicon [31–39]. Detecting hazardous molecules, such as pesticides, explosives, and chemical threats (nerve agents) using flexible SERS substrates is the central aspect of the sensing field because of its simple sample collection from any rough surface [36,40]. Filter paper (FP) SERS substrates are rigorously investigated for the detection of hazardous dye molecules such as crystal violet (CV) and malachite green (MG) on fish [41], pesticides on vegetables, dals [42], fruit surfaces [43], and explosives on rough surfaces [44–45]. In the last few years, our group has been continuously working on developing a flexible SERS substrate for the detection of various types of hazardous molecules: aggregated Ag and Au NPs on filter paper [46], Au NPs on electrospun polymer nanofibers [33], and alloy Ag/Au NPs on filter paper [44]. However, the size-dependent SERS performance of NPs over time needed to be investigated, and the optimization of substrates, depending on their stability over time, was aimed to be studied.

This study investigated the impact of 355, 532, and 1064 nm wavelengths on picosecond laser ablation of silver, gold, and silver/gold alloy samples within two distinct liquid media: distilled water (DW) and aqueous NaCl solution. Significant variations in the productivity and size of NPs were observed across different wavelengths and media. Subsequently, a flexible SERS substrate was developed by depositing 18 types of NPs produced onto a filter paper. It was found that substrates containing NPs generated at 1064 nm laser wavelength exhibited prominent performance, characterized by higher yield and larger particle size. Stability tests revealed that NPs in the electrolyte (NaCl) solution displayed a quicker decline in SERS signal than those obtained in DW, despite satisfactory initial signal strengths. However, gold NPs in DW demonstrated optimal long-term stability, maintaining uniform SERS intensities over 60 days. Further, optimized SERS substrates were tested with different Raman excitations to highlight the critical role of molecular resonance absorption. Specifically, an excitation wavelength of 325 nm proved to be the most effective for detecting methyl salicylate and DMMP, underscoring the importance of selecting an appropriate excitation wavelength for enhanced molecular detection.

## Experimental

### Materials

Dimethyl methyl phosphonate (DMMP, C_3_H_9_O_3_P, 98% pure); methyl salicylate (MS, C₈H₈O₃, 99% pure), methylene blue (MB, C_16_H_18_ClN_3_S), and thiram (C_6_H_12_N_2_S_4_) were purchased from Sigma-Aldrich. All chemicals were of analytic grade and used for cleaning and diluting the samples. The laser ablation samples were 99% pure and had a thickness of 1 mm.

#### Synthesis of nanoparticles by laser ablation in liquid

Initially, silver, gold, and silver/gold (Ag_50_Au_50_) alloy targets (99%) were obtained from a local market and cut into 1 cm × 1 cm pieces. The targets were thoroughly cleaned in an ultrasonic bath using ethanol, acetone, and DW for 10 min. After washing, the targets were fixed at the bottom of the glass beaker filled with 5 mL of DW and aqueous NaCl solution (1 mM). The setup was mounted on the motorized X–Y translation stage (Newport) connected to a motion (ESP-300) controller. The Ag/Au/Ag_50_Au_50_ targets were ablated using a ps laser (Nd: YAG, EKSPLA PL2351), delivering ≈30 ps pulses at a wavelength of 1064, 532, and 355 nm at a 10 Hz repetition rate. For each wavelength, meticulous alignment of the laser beam was ensured using mirrors explicitly chosen for their optimal performance within the respective wavelength ranges. The laser beam was guided towards the processing region by mirrors and eventually focused on the sample surface by a lens of focal length (*f*) ≈10 cm at normal incidence. Ablation experiments were performed at a pulse energy of 12 mJ with the corresponding laser fluence of ≈30 mJ/cm^2^. The moving target was irradiated using a separation of 50 μm between two adjacent lines at a translation speed of 1 mm/s along both directions. The total laser-processing area on the target surface was typically ≈25 mm^2^. The ablation experiments lasted for ≈80 min in each case. The ablation process was executed by varying laser wavelengths (355, 532, and 1064 nm) and keeping all the other parameters constant, such as laser pulse energy, repetition rate, quantity of liquid, and focusing conditions. To avoid confusion, the names of the ablated samples and their descriptions are provided in Table 1.

**Table 1:** Summary of the sample descriptions and laser parameters used in this study.

Nanoparticles (NPs)	LaSiS Wavelength					

	355 nm		532 nm		1064 nm	

Solvent	DW	NaCl	DW	NaCl	DW	NaCl
Ag	AgD1	AgN1	AgD2	AgN2	AgD3	AgN3
Au	AuD1	AuN1	AuD2	AuN2	AuD3	AuN3
Ag50Au50	AgAuD1	AgAuN1	AgAuD2	AgAuN2	AgAuD3	AgAuN3

#### Preparation of filter-paper-based flexible SERS and SERS measurements

Flexible substrates were fabricated using Whatman FP as the base material, which was cut into small squares of 1 cm^2^ each. Subsequently, metal and alloy NPs synthesized via laser ablation (encompassing 18 distinct samples) were applied to the FP through a straightforward drop-casting method and then allowed to dry at room temperature. Following this, the analyte of interest was also applied onto the substrate using drop casting. This process took approximately 20 minutes.

#### Characterization techniques

The absorption studies were conducted by placing 3 mL of the colloidal solution in a 1 cm quartz cuvette and using a UV–visible absorption spectrometer (PerkinElmer, LAMBDA 750) within the 300–800 nm wavelength range. The distribution of NPs on a FP was analyzed using the INCA software with a field-emission scanning electron microscope (FESEM, Carl Zeiss Ultra 55). Samples were prepared by drop casting 10 µL of NPs onto a FP, followed by sputtering a thin conductive layer of gold onto the FP to facilitate lower magnification imaging due to the nonconductive nature of the FP substrate. FESEM energy-dispersive X-ray spectroscopy (EDX) mapping investigations were conducted on Ag/Au alloy NPs deposited on a Si substrate by drop casting 10 µL to avoid confusion in the data caused by the Au coating. Transmission electron microscopy (TEM) was performed with a JEM-2100F (JEOL, Japan). TEM grids were prepared by drop casting 2 µL of NPs onto the grids. Raman/SERS spectra were collected using a portable Raman spectrometer (B&W Tek) with an excitation wavelength of 785 nm, 10 mW of laser power, 5 s of collection time, and three accumulations. The laser beam spot size on the sample was ≈100 µm. Wavelength-based SERS measurements were performed using a micro-Raman spectrometer (Horiba-Scientific), and 325, 532, and 633 nm laser excitation wavelengths with the same input laser power of ≈1 mW, 5 s of acquisition time, and three accumulations, and these parameters were maintained in all the measurements. For the focusing conditions, a 50× objective was used for the visible wavelengths (532 and 632 nm), while a 40×-NUV objective was employed for the 325 nm wavelength. A baseline correction using the Origin software was applied to all recorded SERS spectra, after which SERS calculations were undertaken.

## Results and Discussion

### Characterization

#### Optical absorption studies of as-synthesized nanoparticles

**Figure 1:**
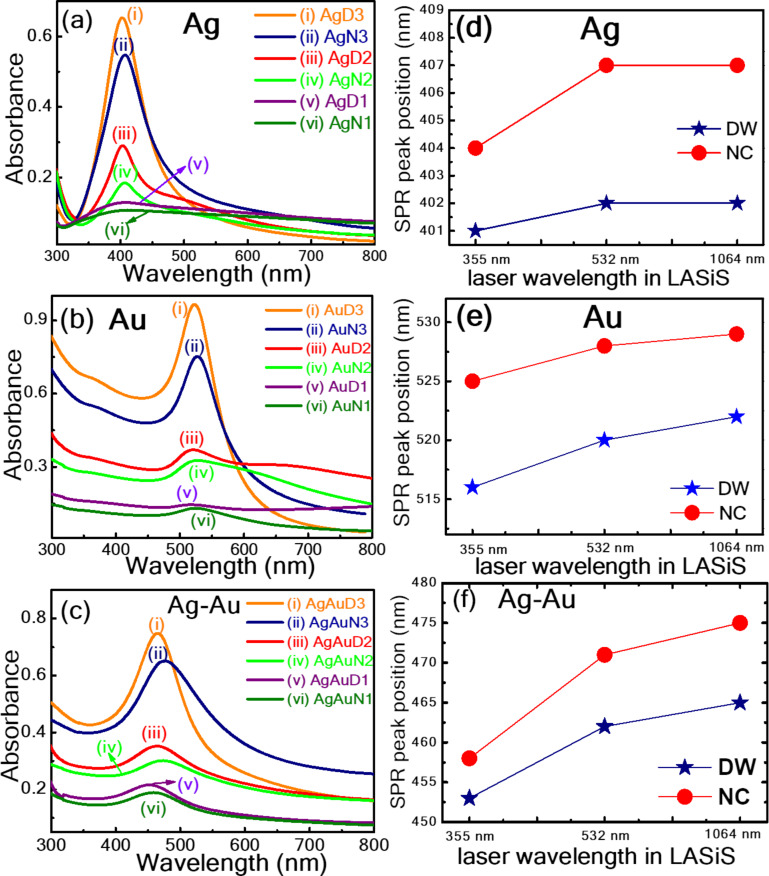
(a)–(c) Absorption spectra of laser-synthesized NPs (a) Ag NPs [AgD1, AgD2, AgD3, AgN1, AgN2, AgN3]; (b) Au NPs [AuD1, AuD2, AuD3, AuN1, AuN2, AuN3]; (c) Ag/Au NPs [AgAuD1, AgAuD2, AgAuD3, AgAuN1, AgAuN2, AgAuN3] obtained at different laser wavelengths (1064, 532, and 355 nm) and different liquids (DW and aqueous NaCl solution). (d–f) The variation of SPR peak position of (d) Ag, (e) Au, and (f) Ag/Au concerning wavelength and surrounding liquid.

The absorbance measurements were carried out on metallic Ag, Au, and Ag/Au NPs prepared using ps LASiS in both DW and aqueous NaCl solution, covering a wavelength range of 300–800 nm. In Figure 1, the optical absorption spectra of (a) Ag, (b) Au, and (c) Ag/Au NP solutions are presented for both environments obtained at 1064, 532, and 355 nm wavelengths in LASiS. All absorption spectra exhibit a distinct single surface plasmon resonance (SPR) absorption peak, indicating the formation of spherical NPs. The SPR peak of Ag/Au alloy NPs lies between the SPR peak positions of pure Ag and Au NPs. Notably, the plasmon bands of NPs obtained at lower wavelengths (355 and 532 nm) are broadened compared to those of NPs fabricated at higher wavelengths (1064 nm). This broadening could be ascribed to the size/shape of the NPs, their aggregation, and variations in size distribution under different laser wavelengths. The NP productivity in the LASiS approach is mainly influenced by laser wavelength irradiation based on the interaction of the material with the incoming beam, including absorption, reflection, and scattering [47–48]. These interactions significantly vary across the wavelengths from 1064 down to 355 nm, with a unique response of different materials at each wavelength [49]. A key to enhancing the yield is choosing the laser wavelength at which the target material has a high absorption rate. This ensures that a greater energy density is transferred to the target, thereby increasing the volume of material ablated. On the other hand, to minimize energy losses during the process, choosing the surrounding media is also an essential factor, which could prevent the absorption of the aqueous solution at a given laser wavelength. This approach helps to achieve a delicate balance between maximizing the absorption in the target material while minimizing energy loss in the surrounding liquid and NPs, thereby enhancing the overall efficiency and yield of the LASiS process [4,50]. The study reported by Shukri et al. [51] pointed out a size reduction in Au NPs from 19 to 12 nm by decreasing the input laser wavelengths, changing from 1064 to 532 nm in DW. Solati et al. [52] observed a significant increase in the mean size of Ag NPs from 13 to 32 nm while ablating the Ag targets in acetone with laser wavelengths of 532 and 1064 nm. Furthermore, the absorption intensity proportionally increases with the increasing wavelength in both DW and NaCl, demonstrating that the yield of NPs is higher at higher wavelengths. Moreover, the absorption of NPs produced in aqueous NaCl solution was lower than that in DW, indicating a higher NP yield in DW. The observed difference in absorption intensities could be attributed to the influence of NaCl in the NP synthesis process. Salts may affect the kinetics of NP formation, leading to size, shape, and aggregation variations, ultimately impacting their optical properties [53]. Also, from absorbance studies, it is observed that the ablation rate is higher for the Au target than for Ag because of the hardness variation observed in an earlier report by Solati et al. [54]. In another study by Bae et al. [55], the authors demonstrated the Ag NP fabrication by varying the surrounding aqueous NaCl solution concentrations between 0 to 20 mM using a nanosecond laser at an excitation of 355 nm. Their study noticed increased Ag NP absorbance while changing the aqueous NaCl solution concentration from 0 to 5 mM, which decreased further. The SPR peaks were blue shifted for NPs obtained in NaCl compared to those obtained in DW, which could be attributed to an increase in the refractive index of the surrounding medium. The SPR shifts at different wavelengths of LASiS within the same environment suggest the variations of NP particle size or size dispersion. The peak shift is mainly correlated with the size and shape of NPs and their surrounding medium. Naderi-Samani et al. [56] reported the synthesis of Ag NPs by nanosecond laser ablation in different aqueous solutions: water, acetone, cetyltrimethylammonium chloride (CTAC), polyvinylpyrrolidone (PVP), and sodium dodecyl sulfate (SDS). Their outcomes revealed that the productivity of Ag NPs was higher in acetone, followed by CTAC, water, PVP, and SDS. The order of NP formation efficiency was reported to be acetone >CTAC >water >PVP >SDS.

#### Morphological studies of as-synthesized nanoparticles

**Figure 2:**
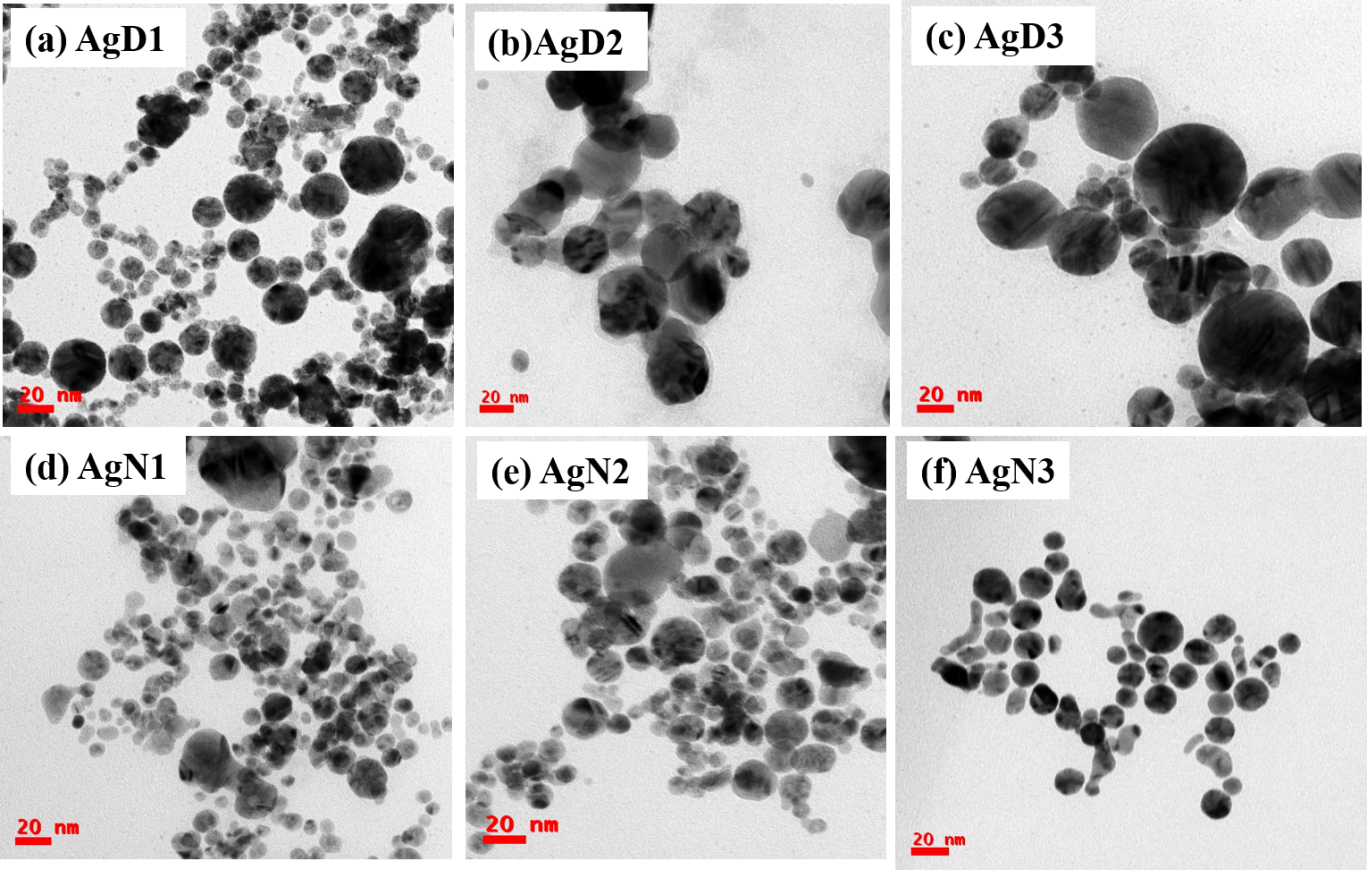
TEM images of Ag NPs: (a) AgD1, (b) AgD2, (c) AgD3, (d) AgN1, (e) AgN2, and (f) AgN3.

Transmission electron microscopy analysis was implemented to study the size and shape of laser-fabricated NPs. Figure 2 shows the TEM images of Ag NPs synthesized in DW at different laser wavelengths: (a) 355, (b) 532, and (c) 1064 nm. It should be noted that the shape of NPs is spherical, and the size distribution of the Ag NPs is strongly dependent on laser wavelength in LASiS. The average size of the NPs was estimated as 12.4 ± 0.3 nm at 355 nm, 23.9 ± 1.0 nm at 532 nm, and 36.3 ± 3.7 nm at 1064 nm, with the size distributions being provided in Supporting Information File 1, Figures S1(a)–(c). It is believed that with increasing wavelength, the NP sizes increase, presumably due to the coexistence of both processes, such as laser ablation and laser fragmentation in liquids at lower wavelengths (i.e., higher energy). The SPR peak is shifted toward a longer wavelength for larger particles, which is evident from the absorption spectra depicted.

Figure 2 depicts the pictures of Ag NPs synthesized using a laser wavelength of (d) 355 nm, (e) 532 nm, and (f) 1064 nm in an aqueous NaCl solution. The average particle size is 8.4 ± 0.4 nm at 355 nm, 13.3 ± 0.5 nm at 532 nm, and 16.5 ± 0.5 nm at 1064 nm, respectively. The size distributions are provided in Supporting Information File 1, Figure S1(d–f). The size of the NPs increases with increasing laser wavelength. It is worth mentioning that a reduction in NP size was noticed more in NPs produced in aqueous NaCl solution than in those fabricated in DW. The size reduction effect observed during ablation in aqueous NaCl solution can be attributed to Cl^−^ ions [55,57]. When a laser ablation process is conducted in the presence of NaCl, the ions in the solution can strongly influence nucleation processes and growth in the generation of the NPs. The presence of electrostatic repulsion among charged NPs generated in an electrolyte solution reduces the average size of the NPs. Rehbock et al. [21] pointed out the size reduction in Au NPs from 30 nm at 3 µM to 7 nm at 500 µM in the presence of aqueous NaCl solution during the ablation. He et al. [58] demonstrated that at higher NaCl (10 mM) concentrations, ZnO NPs exhibited coalescence, increasing NPs size in comparison to those obtained in DW.

**Figure 3:**
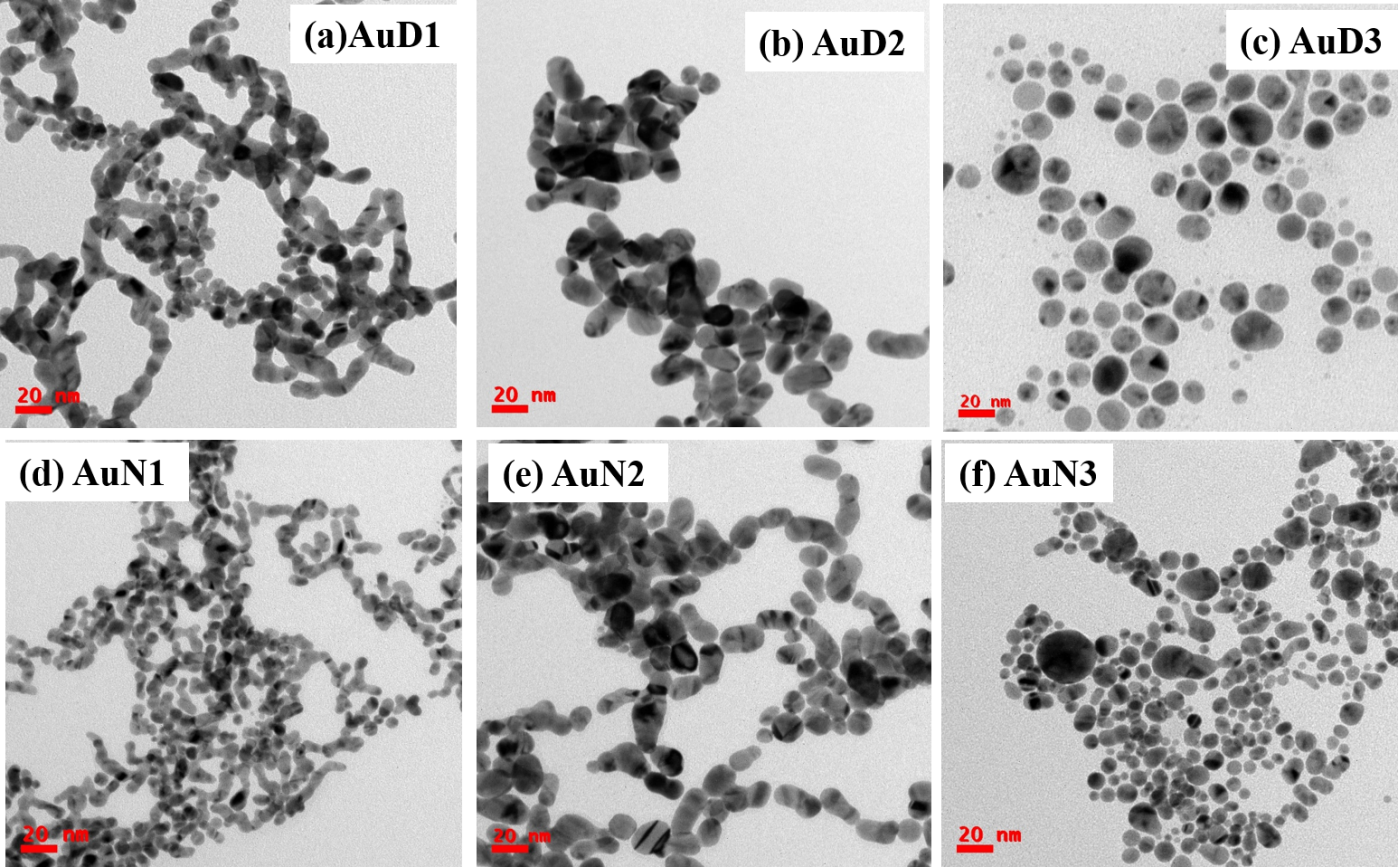
TEM images of Au NPs: (a) AuD1, (b) AuD2, (c) AuD3, (d) AuN1, (e) AuN2, and (f) AuN3.

In Figure 3, TEM images depicting as-synthesized Au NPs under different incident laser wavelengths (i.e., (a) 355, (b) 532, and (c) 1064 nm) in DW are shown. At 355 nm, a distinctive nanochain morphology linking spherical NPs was evident, contrasting with the separated spherical morphology. The prevalent interaction at 355 nm with the liquid phase was more influential than the NP production, resulting in particles with fragmented shapes [6]. This could be accredited to the more vital interaction of the lower wavelength with the liquid than with the submerged solid target. During ablation, the maximum energy dedicated to the formerly generated NPs resulted in further fragmentation of NPs and fusion rather than in target ablation. This phenomenon led to lower NP production at 355 nm compared to that at 532 and 1064 nm laser wavelengths, this was also reflected in the absorption spectra. Consequently, there is a tendency for NP agglomeration and chain formation, which is evident from the TEM pictures depicted in Figure 3. As the wavelength increases, ablation becomes more efficient, breaking the chains and creating smaller, separated spherical NPs. The mean sizes of the NPs synthesized at the three selected wavelengths are estimated to be approximately 9.5 ± 0.1 nm at 355 nm, 15.6 ± 0.1 nm at 532 nm, and 19.7 ± 0.7 nm at 1064 nm. The size distributions are provided in Supporting Information File 1, Figures S2(a)–(c). Notably, the mean diameter of NPs is smaller when generated at lower wavelengths compared to those produced at higher wavelengths.

**Figure 4:**
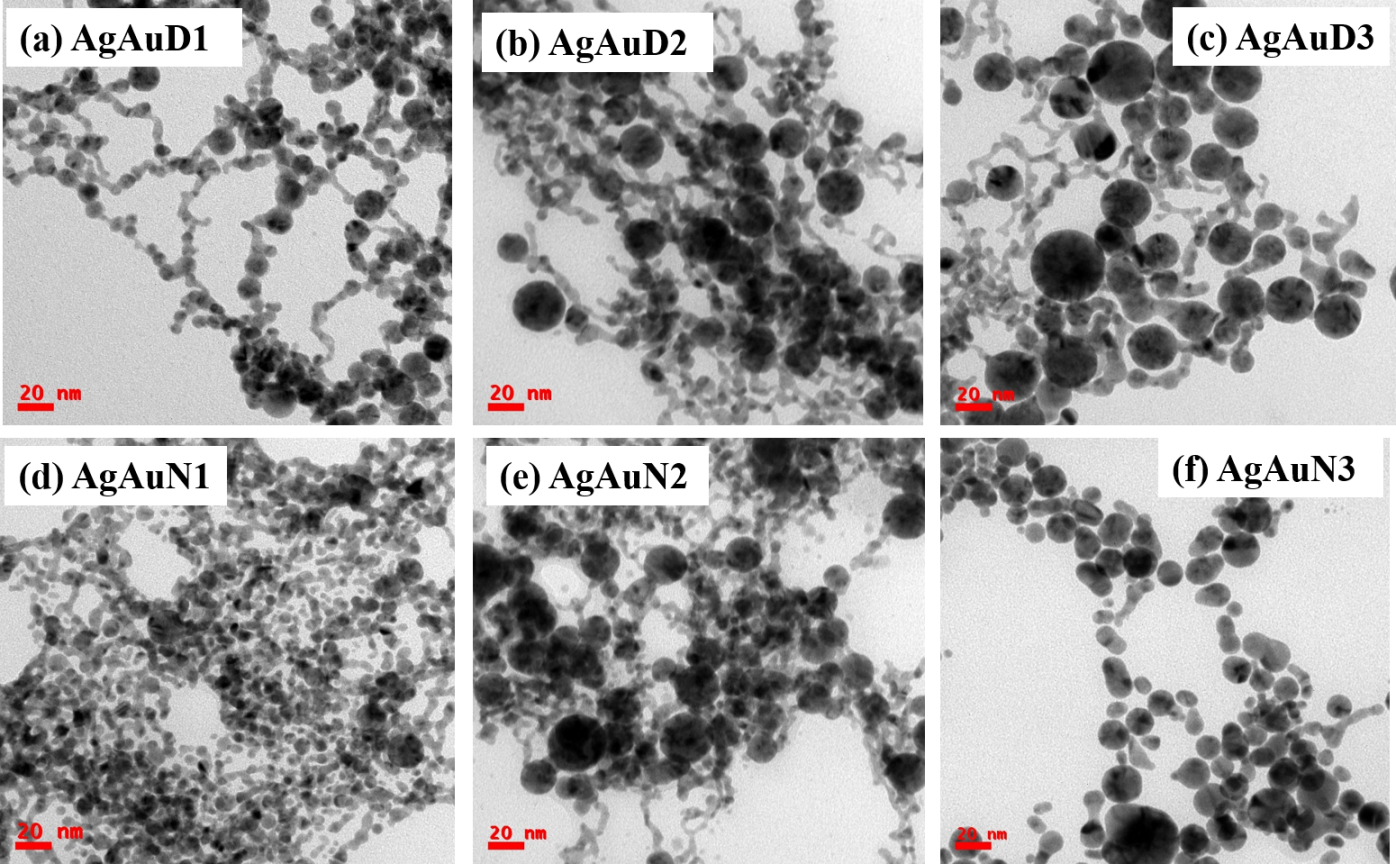
TEM images of Ag/Au NPs: (a) AgAuD1, (b) AgAuD2, (c) AgAuD3, (d) AgAuN1, (e) AgAuN2, and (f) AgAuN3.

Figure 3 displays TEM images depicting the synthesis of Au NPs under different laser wavelengths: (d) 355, (e) 532, and (f) 1064 nm in aqueous NaCl solution. Furthermore, the presence of NaCl in the surrounding medium significantly influences NP size, leading to size reduction. The mean sizes of the NPs in the presence of NaCl are estimated as 7.0 ± 0.5 nm at 355 nm, 11.4 ± 0.6 nm at 532 nm, and 12.6 ± 0.1 nm at 1064 nm. The size distributions are provided in Supporting Information File 1, Figures S2(d)–(f). This size reduction aligns with the observed behavior of nanochains at lower wavelengths and separated NPs at higher wavelengths. Figure 4 shows TEM images of Ag/Au alloy NPs under different laser wavelengths: (a,d) 355, (b,e) 532, and (c,f) 1064 nm in DW and aqueous NaCl solution, respectively. The mean sizes of the NPs in DW and NaCl are 24.9 ± 3.3 and 15.2 ± 0.2 nm at 355 nm, 12.7 ± 1 and 8.2 ± 0.3 nm at 532 nm, and 6.5 ± 0.1 and 5.8 ± 0.1 nm at 1064 nm, respectively. The NP size distributions are provided in Supporting Information File 1, Figure S3.

**Figure 5:**
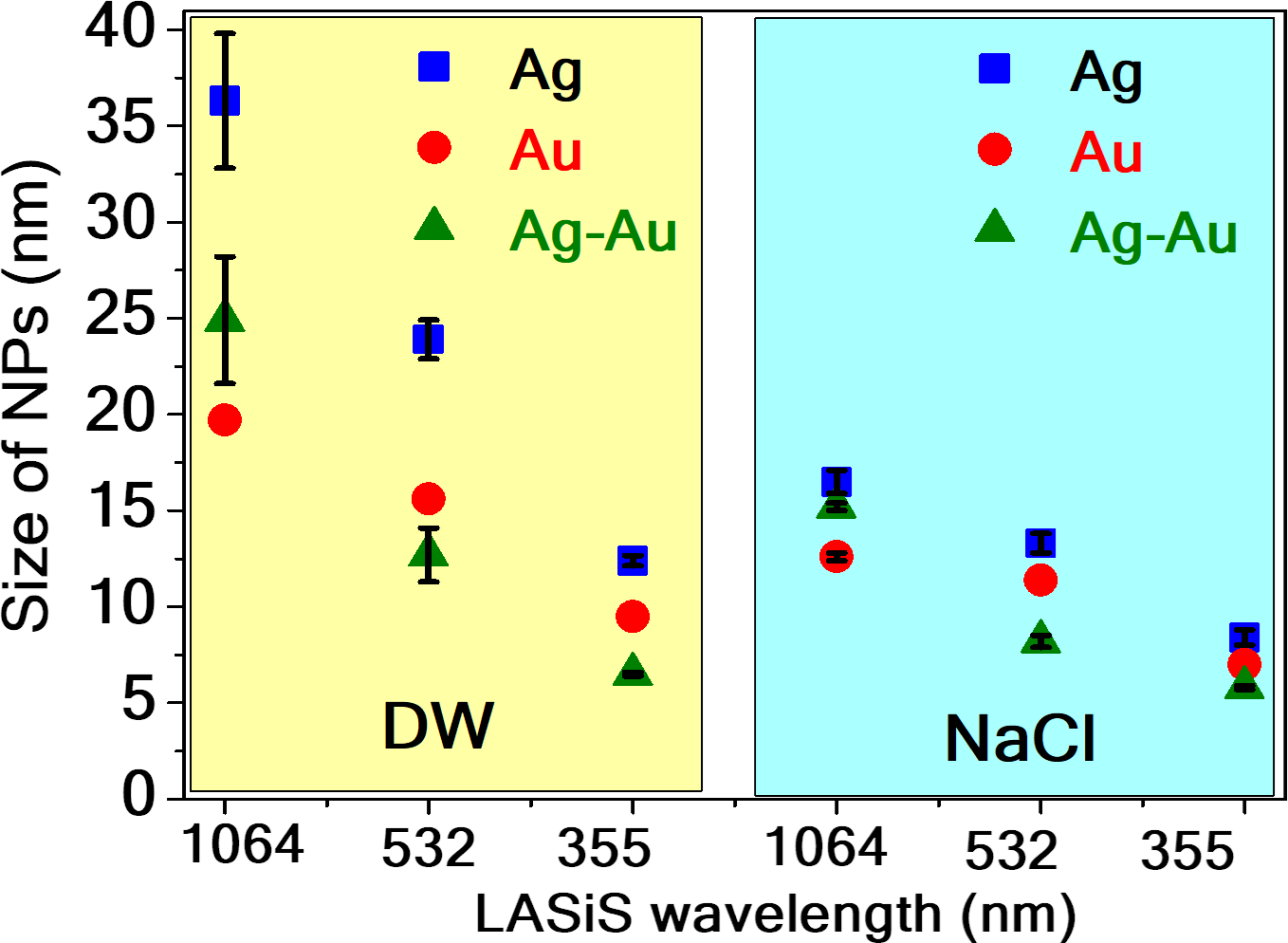
The variation in sizes of NPs at different wavelengths and environments. The left panel displays the size variation for Ag, Au, and Ag/Au in DW, while the right panel illustrates size variation in an aqueous NaCl solution.

The variation in the sizes of Ag, Au, and Ag/Au NPs obtained in DW and aqueous NaCl solution at different wavelengths during the LASiS process is illustrated in Figure 5. It is noted that the NP size increased while the laser wavelength was increased. Size reduction was observed when the aqueous NaCl solution was used as a surrounding medium instead of other liquids (DW). In this case, the size increases with an increasing wavelength. The diameters of colloids prepared at a laser wavelength of 1064 nm in DW are greater than 20 nm. However, NPs obtained in an aqueous NaCl solution at a laser wavelength of 355 nm showed nanochain features with diameters smaller than 10 nm.

#### Topographical studies of filter paper-loaded nanoparticles

**Figure 6:**
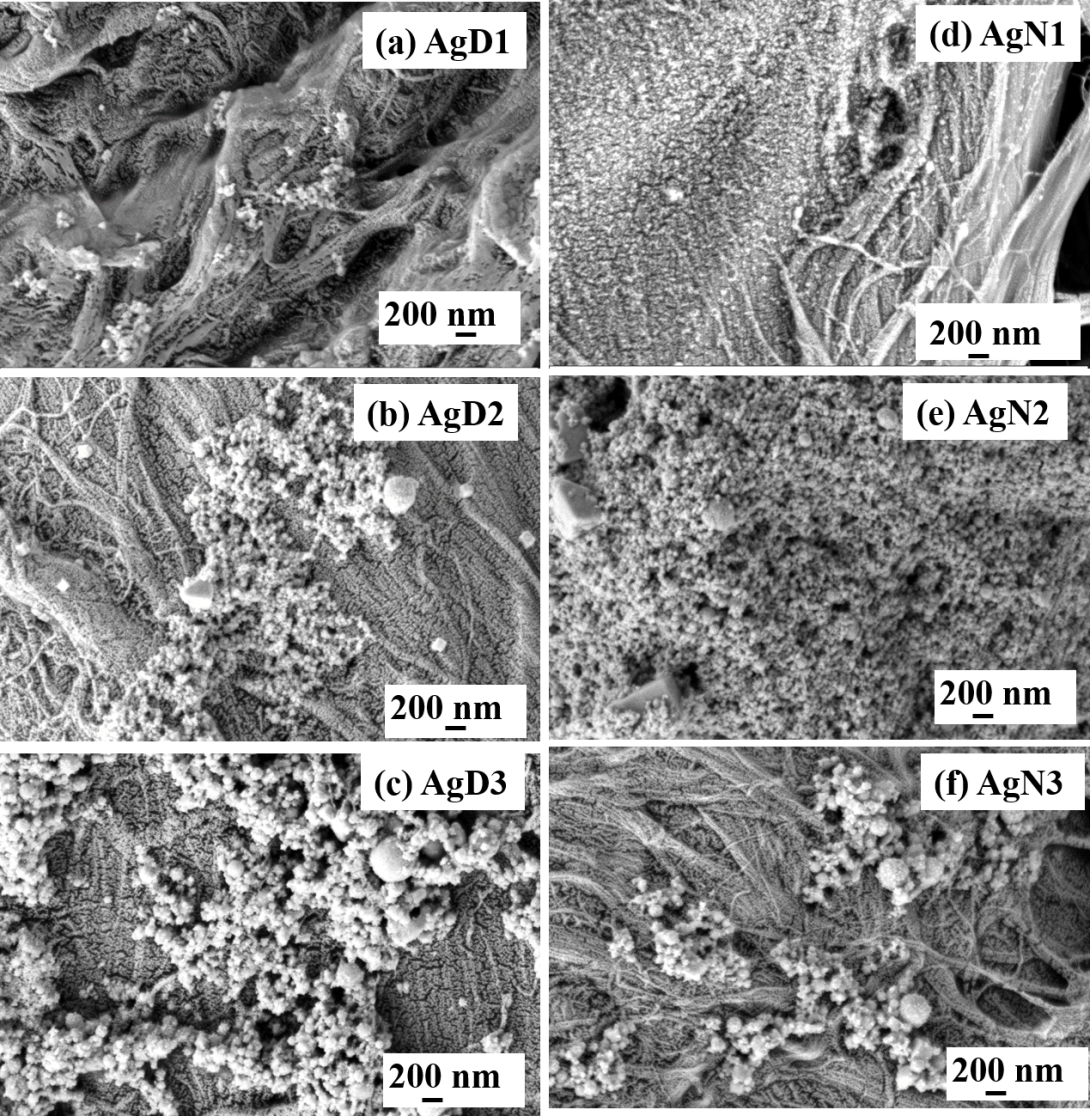
Panels (a)−(c) display FESEM images of a filter paper loaded with Ag NPs obtained in DW: (a) AgD1, (b) AgD2, and (c) AgD3. Panels (d)−(e) display FESEM images of a filter paper loaded with Ag NPs obtained in aqueous NaCl solution: (d) AgN1, (e) AgN2, and (f) AgN3 at wavelengths of 355, 532, and 1064 nm, respectively.

**Figure 7:**
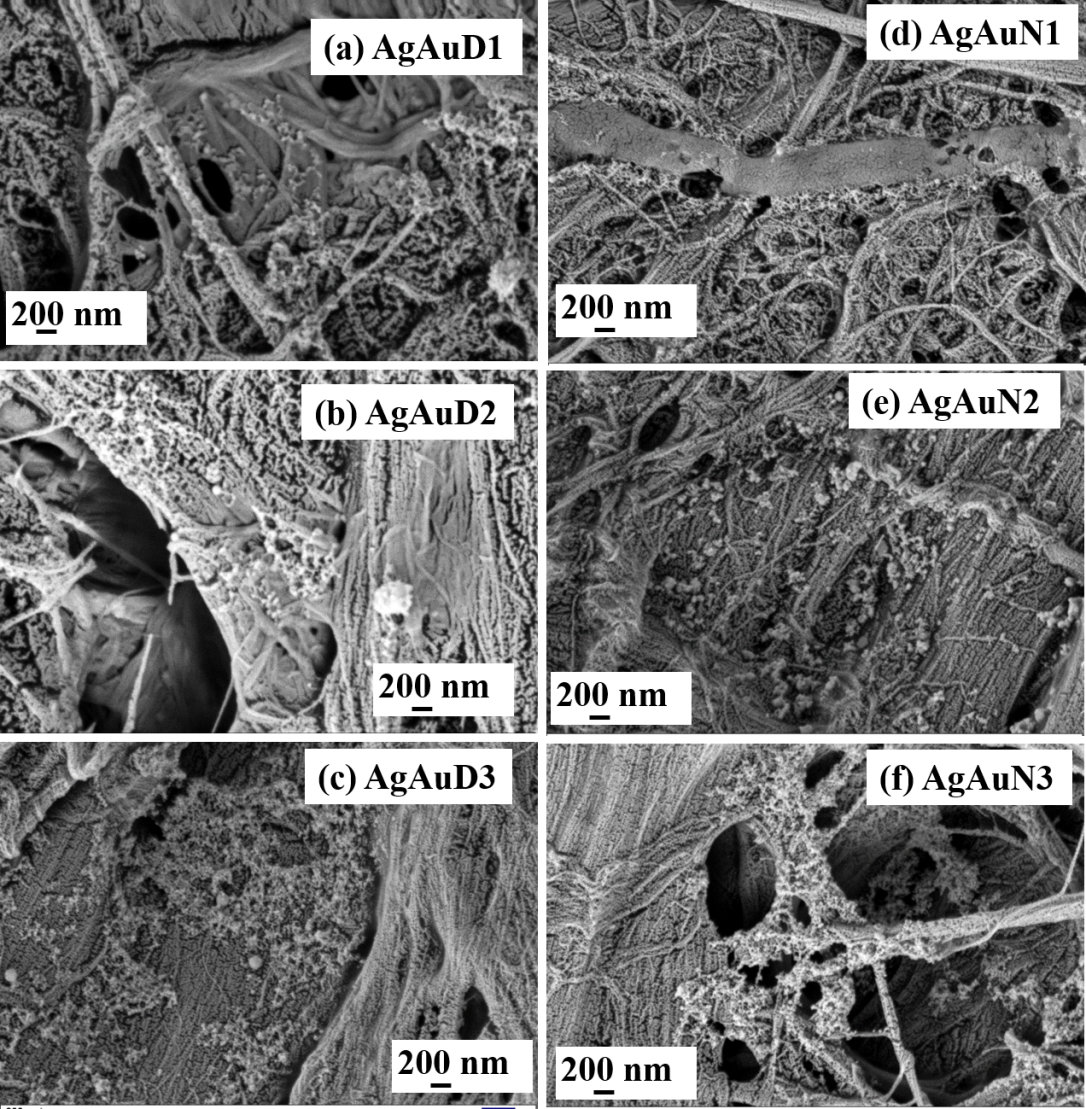
Panels (a)−(c) display FESEM images of a filter paper loaded with Ag/Au NPs obtained in DW: (a) AgAuD1, (b) AgAuD2, and (c) AgAuD3. Panels (d)−(e) display FESEM images of a filter paper loaded with Ag NPs obtained in aqueous NaCl solution: (d) AgAuN1, (e) AgAuN2, and (f) AgAuN3 at a wavelength of 355, 532, and 1064 nm, respectively.

The morphology and distribution of ps laser-fabricated NPs on filter paper were investigated using FESEM. The FESEM images reported in Figure 6 depict a filter paper surface grafted with Ag NPs, categorized as: (a) AgDW1, (b) AgD2, (c) AgD3, (d) AgN1, (e) AgN2, and (f) AgN3. Similarly, a FP loaded with Ag/Au NPs is illustrated in Figure 7, categorized as: (a) AgAuD1, (b) AgAuD2, (c) AgAuD3, (d) AgAuN1, (e) AgAuN2, and (f) AgAuN3. It is evident from the FESEM images that the concentrations of loaded NPs were different, possibly due to the differences in their loading and the effect of NPs yield obtained at different laser wavelengths. Furthermore, recorded FESEM-EDX data confirms the occurrence of Ag, Au, Na, Cl, C, and O elements on the FP-AgAuN3 substrate, provided in Supporting Information File 1, Figure S4. To confirm the presence of Ag and Au in alloy NPs, an EDX mapping investigation was conducted on AgAuD3 NPs coated on a Si substrate. The color map image of a single alloy NP with a squared area was represented in Supporting Information File 1, Figure S5. It is evident from the figure that individual NPs encompassed both Ag and Au elements. The detailed mechanism underlying the formation of alloy NPs was elucidated in our previous study [44]. Earlier studies suggested that the NP yield gradually increases with increasing laser wavelength. Notably, we could achieve a higher yield of NPs at higher wavelengths, resulting in a higher concentration of NPs on the filter paper surface. Additionally, larger NPs were observed at higher wavelengths than at lower wavelengths, which is also evident from TEM image analysis, as discussed above. A similar trend was noticed in the case of Au NP distribution on FP, as detailed in Supporting Information File 1, Figure S6.

#### SERS measurements from filter paper-loaded nanoparticles

**Figure 8:**
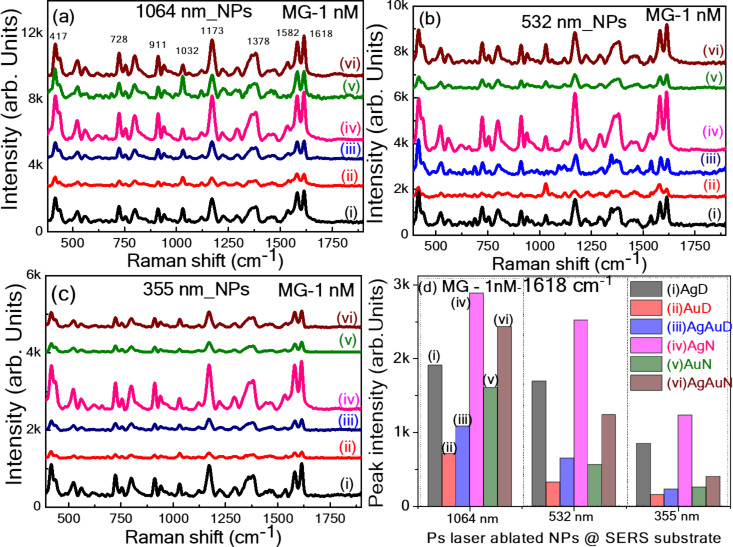
The SERS spectra of MG (1 nM) using filter paper loaded with ps laser ablated NPs (i) AgD, (ii) AuD, (iii) AgAuD, (iv) AgN, (v) AuN, and (vi) AgAuN at (a) 1064, (b) 532, and (c) 355 nm, (d) Intensity histogram of the prominent SERS peak at 1618 cm^−1^ (using a portable Raman spectrometer at an excitation wavelength of 785 nm).

Initially, a portable Raman spectrometer with a fixed excitation wavelength of 785 nm was employed, demonstrating the superior performance of flexible SERS substrates by depositing all the synthesized NPs onto a filter paper. This combination offers practical real-time onsite application capabilities, allowing for immediate and nondestructive analysis in the field, making it ideal for rapid screening and preliminary investigations. The portable spectrometer is user friendly, compact, and the lightweight design ensures minimal sample preparation and ease of transport, providing flexibility in diverse application scenarios. To capitalize on the advantages of a flexible substrate in the sensing field, filter paper was chosen as a base to host laser-ablated NPs [59]. The SERS performance of NP-loaded paper-based SERS substrates was assessed by choosing malachite green (MG) as a Raman reporter molecule. The SERS spectra reported in Figure 8a–c illustrate the prominent Raman bands of MG at a 1 nM concentration recorded from filter paper loaded with NPs, namely, Ag, Au, and Au NPs in DW and aqueous NaCl solution fabricated at three different wavelengths: 1064, 532, and 355 nm. Figure 8d displays the intensity variation of a prominent 1618 cm^−1^ MG peak recorded from all substrates. The location of Raman peaks of MG noticed in our study coincides with the studies demonstrated earlier [60]. The leading characteristic bands found at 1618 cm^−1^ are assigned to phenyl-N and C–C stretching.

**Figure 9:**
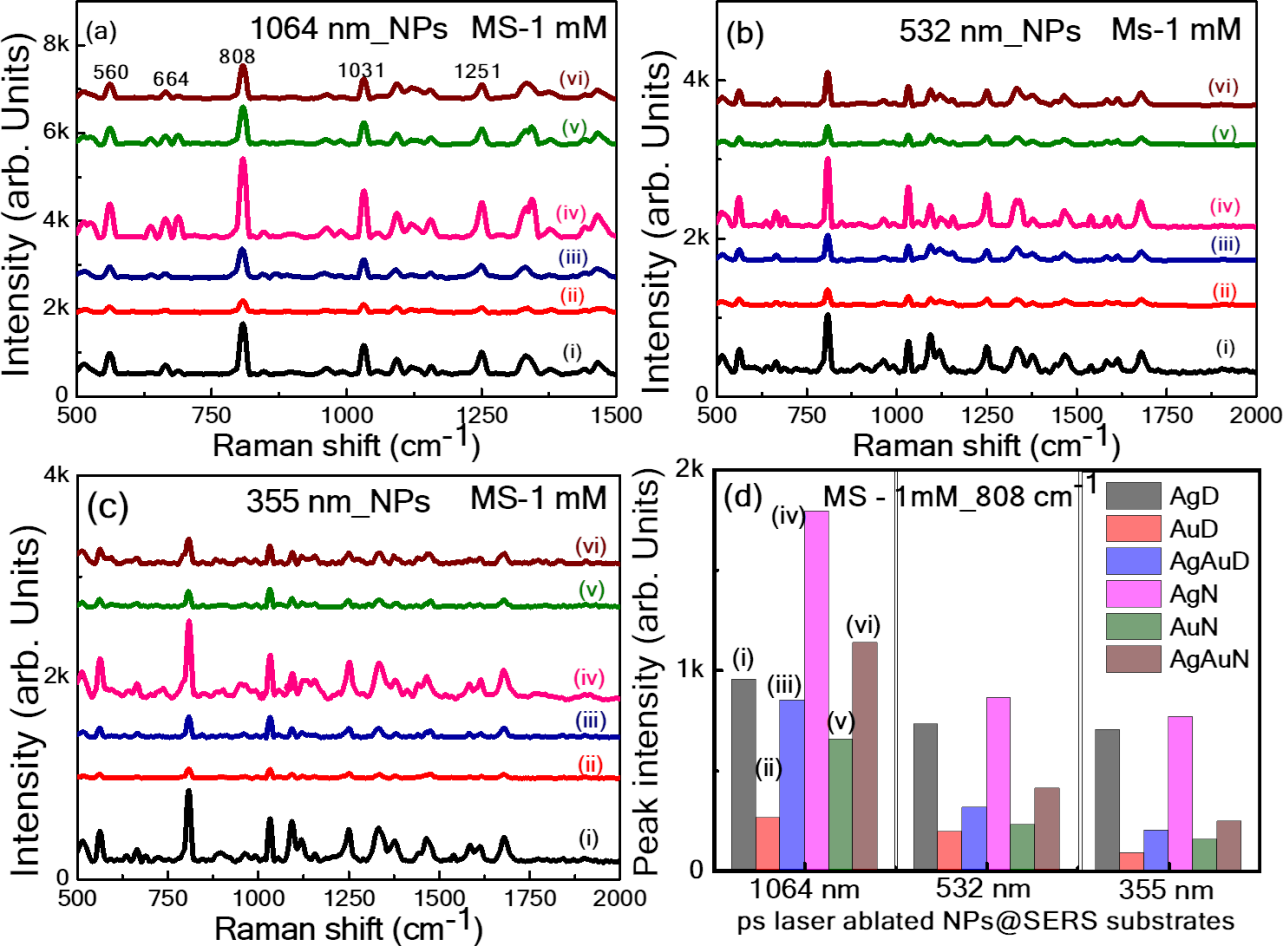
The SERS spectra of methyl salicylate (1 mM) using filter paper loaded with ps laser-ablated Ag, Au, and Ag/Au NPs in DW and aqueous NaCl solution at (a) 1064, (b) 532, and (c) 355 nm. (d) Intensity histogram of the prominent peak at 808 cm^−1^ from all 18 different FP substrates (using portable Raman spectrometer at an excitation of 785 nm).

Similarly, SERS investigations were extended to detect the pesticide molecule thiram (10 µM) on all Ag, Au, and Ag/Au NP-loaded filter paper substrates. The obtained data is provided in Supporting Information File 1, Figures S7a–S7c using LASiS at 1064, 532, and 355 nm, respectively. All peaks align well with previous reports. The central characteristic peak at 1368 cm^−1^ was considered for evaluating performance, as illustrated in the performance histogram shown in Supporting Information File 1, Figure S7d. Further, similar studies are continued with methyl salicylate as a Raman reporter. Figure 9a–c present the SERS spectra of methyl salicylate (1 mM) collected from filter paper loaded NPs substrates obtained at laser wavelengths of (a) 1064, (b) 532, and (c) 355 nm. Figure 9d shows the Raman intensity variation of the central characteristic peak at 808 cm^−1^. From the SERS measurements, we could point out that the filter paper grafted with Ag NPs was achieved in aqueous NaCl solution, demonstrating superior enhancement of AgAu alloy and Au NPs fabricated in DW and NaCl loaded on filter paper. Notably, the FP loaded with AgN3 NPs demonstrated superior SERS enhancement among all Ag NP-based substrates. This can be attributed to several factors, including (i) the plasmonic performance of Ag, (ii) a large number of NPs loaded onto the filter paper (resulting in a high yield of NPs at 1064 nm), (iii) the presence of ions from NaCl which can lead to ion-enhanced SERS effects [37,46,61].

**Figure 10:**
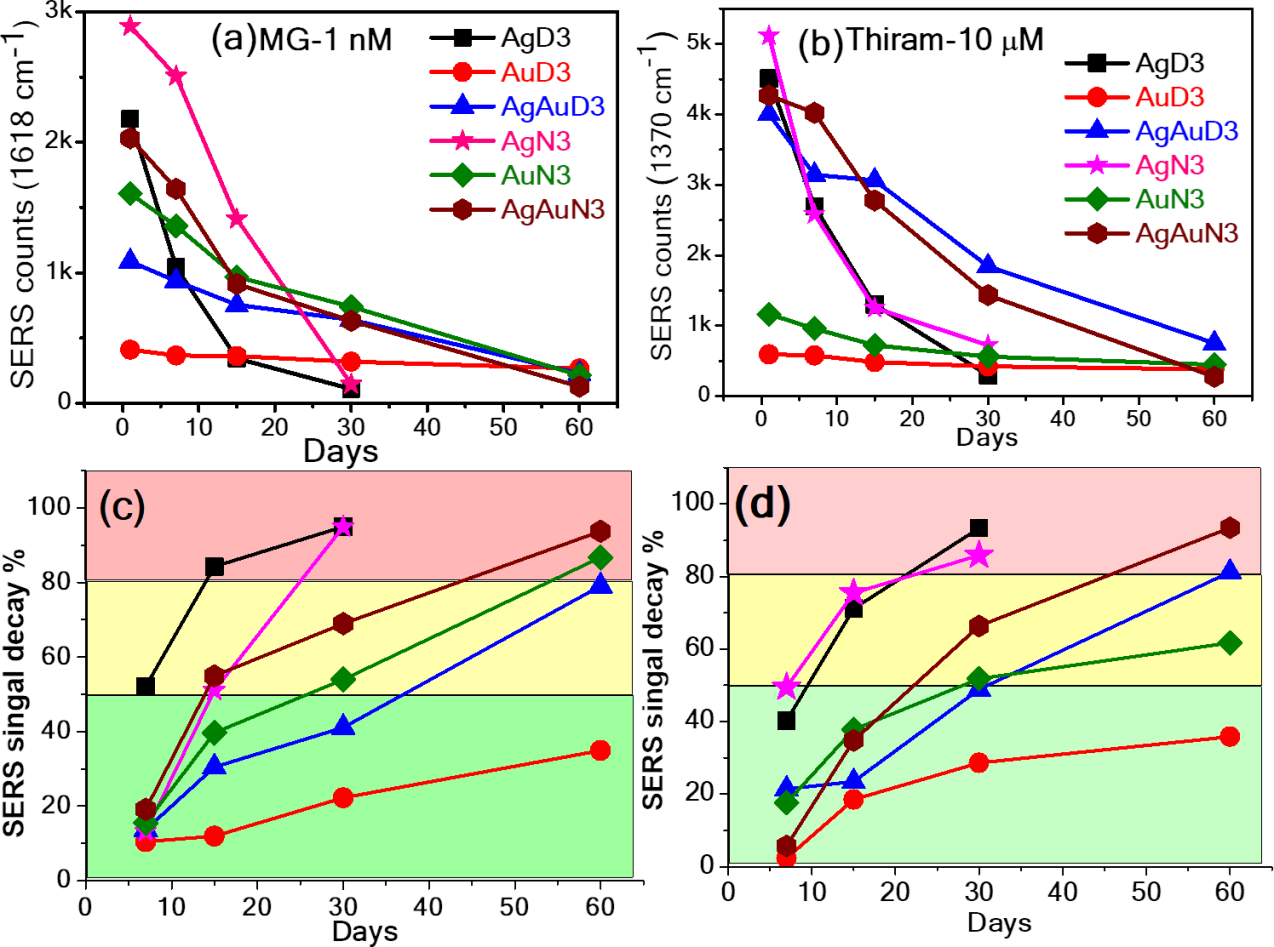
Stability of paper-based SERS substrates: (a) MG (1 nM) and (b) thiram (10 µM). Prominent peak intensity variation (c) and (d) % signal decay as a function of number of days (up to 60) using AgD3, AgAuD3, AuD3, AgN3, AgAuN3, AuN3 NPs loaded filter paper.

The investigations further focused on assessing the stability of a SERS substrate over 60 days. We carried out systematic SERS measurements on different days, and their SERS intensities are compared concerning the days for the substrates, namely AgD3, AgAuD3, AuD3, AgN3, AgAuN3, and AuN3. The evolution of signal intensities over the 60 days is graphically depicted in Figure 10a and Figure 10b, revealing distinctive decay patterns of MG and thiram on the 7th, 15th, 30th, and 60th days. Notably, freshly prepared (on the first day) SERS substrates such as AgD3 and AgN3 exhibited prominent enhancement in Raman signals compared with other substrates, while AuD3 and AuN3 substrates displayed the lowest SERS signal. Decay percentages were calculated to assess the substrate performance. Figure 10c and Figure 10d visually represent the decay percentages over time. On the 30th day, Ag NPs SERS performance decreased by 95%, while Au NPs exhibited variations from ≈10% (7th day) to ≈35% (60th day).

The graphical representation of categorized substrates into three areas based on decay percentages (i.e., green for up to 50%, yellow for 50–80%, and pink for 80–100%) are shown in Figure 10. Substrates, namely AuD3, AgAuD3, AuN3, and AgAuN3 remained stable in the yellow region for up to one month. Notably, the AuD3 substrate showed consistent performance with a decay percentage of 35% over 60 days. Ag/Au alloy NPs often display better properties than those of pure Ag and Au counterparts. Combining Ag and Au can lead to superior plasmonic properties derived from Ag, improved stability attributed to Au, and increased SERS efficiency, making them particularly advantageous for long-term applications with heightened sensitivity. These enhancements are due to synergistic effects, where the integrated properties of the metals surpass those of the individual elements. In the initial days, the presence of NaCl led to an increase in the SERS signal; however, over a longer duration, the NPs in DW showed better SERS intensity than those in aqueous NaCl solution. Jiang et al. [62] investigated the SERS performance of Ag NPs in detecting CV molecules. They found that bare Ag NPs exhibited diminishing performance over three weeks. However, a substrate composed of single atomic layer nanocellulose–Ag NP hybrids maintained nearly constant performance for 35 days. Zhang et al. [63] reported that after undergoing vacuum storage for seven days, the SERS intensity of CV at 1620 cm^−1^ detected on AgNP-120@BNC did not significantly decrease. The SERS intensity of CV detected on the AgNP-120@BNC substrate remained at 91% of its original intensity after seven days of vacuum storage.

#### Effect of Raman excitation wavelength on SERS measurements

The enhanced stability of AuD3 NPs loaded on filter paper regarding SERS performance was further investigated, particularly in hazardous chemical molecules such as methyl salicylate (1 mM) and dimethyl methyl phosphonate (1 mM). Methyl salicylate and dimethyl methyl phosphonate are critical chemical warfare agent (CWA) simulants, posing a significant threat to global security. Detecting these molecules is essential for security reasons, and various detection methods are currently under investigation [64–67]. One promising method for practical application is using flexible SERS substrates as sensing platforms [68].

**Figure 11:**
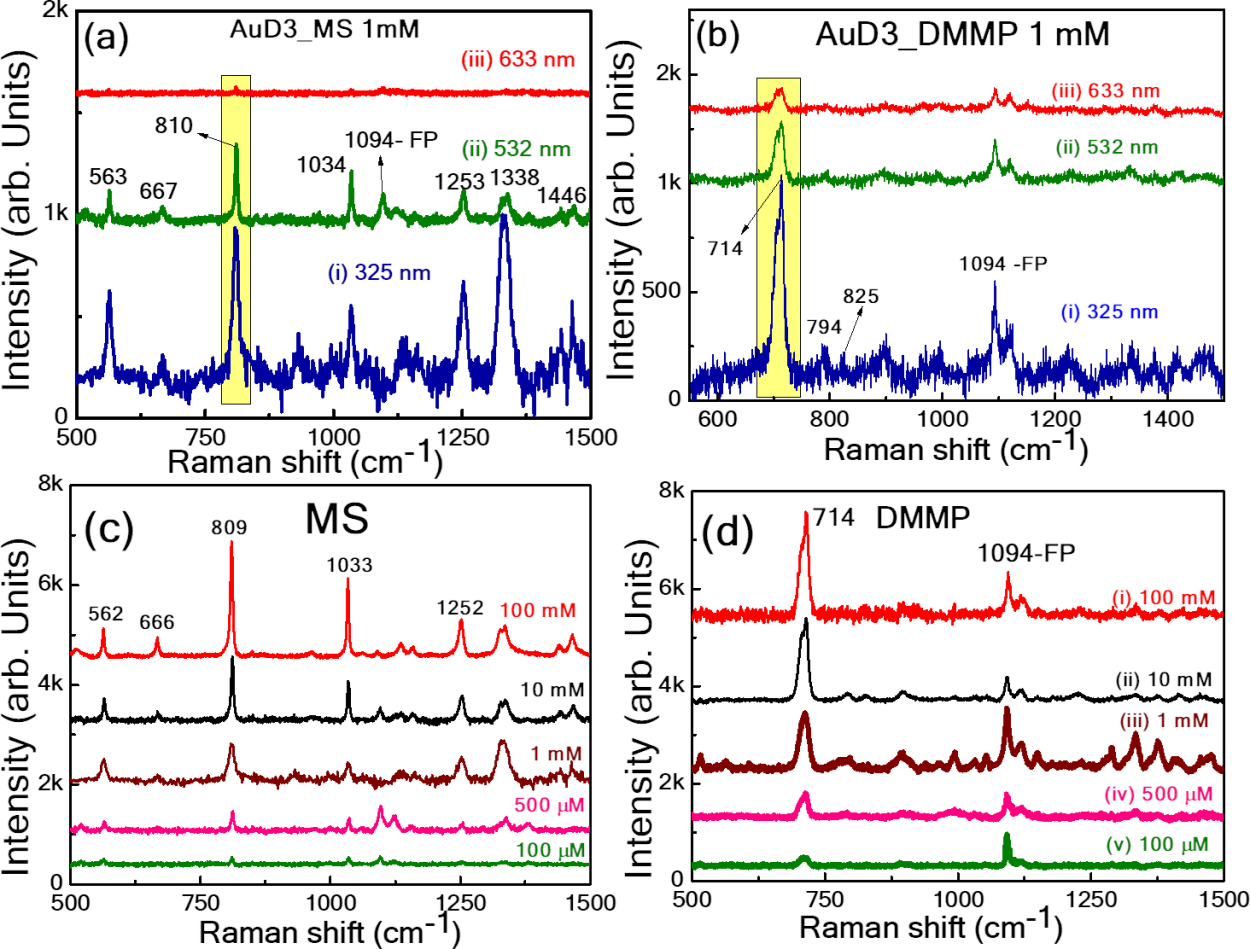
The SERS spectra of (a) MS (1 mM) and (b) DMMP (1 mM) at different Raman laser excitations: 633, 532, and 325 nm. Concentration-dependent SERS spectra of (c) MS (100 mM to 100 µM) and (d) DMMP (100 mM to 100 µM) at an excitation of 325 nm using the AuD3 substrate.

This study explored the impact of different Raman excitation wavelengths, specifically 325, 532, and 633 nm. The prominent peaks of MS (810 cm^−1^) and DMMP (710 cm^−1^) molecules were observed in all cases, shown in Figure 11a and Figure 11b, respectively. The peak at 810 cm^−1^ is due to the stretching vibration of C–H [69], and the peak at 710 cm^−1^ for DMMP is due to the combined vibrational mode, including the symmetrical stretching of the two single P–O bonds and the P–C bond [70]. The peak positions and their assignment of MS and DMMP are provided in Supporting Information File 1, Tables S8 and S9, respectively. Li et al. [69] detected MS (10^−4^ M) by SERS using a Raman excitation of 532 nm. Huang et al. [70] described the identification of DMMP (1 g/L) residues from an irregular surface using Ag NPs grafted cotton swabs via simple swabbing with a laser excitation of 532 nm. Lafuente et al. [71] also reported the detection of DMMP (1.2 ppm V) in the vapor phase using Glass_Ag_Au NPs, 3D fractal microstructure substrates developed by corner lithography and anisotropic wet etching of silicon using the 785 nm as the Raman excitation. When UV excitation was utilized during the measurements, the Raman peak intensities were notably higher for both MS and DMMP molecules at 124 and 152 ppm. This can be attributed to the resonance absorption of these molecules with laser excitation, which increased Raman signal intensity. The reproducibility of the substrate at these different wavelengths in detecting MS was also verified by collecting Raman spectra from more than 15 different locations. The obtained relative standard deviations (RSDs) were 20%, 10%, and 9% for Raman excitations at 633, 532, and 325 nm, respectively. The calculated histograms are provided in Supporting Information File 1, Figure S10a–c.

The sensitivity of the optimized FP SERS substrate was further examined at 325 nm with various concentrations of MS and DMMP ranging from 100 mM to 100 µM, as depicted in Figure 11c and Figure 11d. The leading prominent peaks of MS at 809 cm^−1^ and DMMP at 714 cm^−1^ were observed at the lowest concentration of 100 µM. The reproducibility of the SERS substrate was also examined with the DMMP molecule at a concentration of 500 µM at 15 different locations on the substrate. The histogram showing the most prominent intensity variation yielded an RSD of ≈6%, as shown in Supporting Information File 1, Figure S10d. Aligholizadeh et al. [72] detected DMMP using a handheld Raman spectrometer with an excitation of 785 nm, and the analytes were measured in a liquid-phase solution. The limit of detection (LOD) achieved for DMMP was 9 mM. Chang et al. [67] utilized a combination of cotton swabs with a Ag NP substrate along with a smartphone application to detect DMMP. They detected DMMP at a concentration of 1g/L (≈8 mM) using a Raman excitation of 532 nm. Wang and co-workers [73] developed a novel method using thin water film confinement to enhance Raman detection of weakly interacting nerve agent simulants, such as DMMP (mM), on SERS substrates using a Raman excitation of 633 nm. Li et al. [74] detected, using a portable Raman device at an excitation of 785 nm, DMMP at a concentration of 100 mg/kg (800 µM) utilizing a Au@ZrO_2_ substrate.

## Conclusion

In conclusion, this study delved into the impact of different laser wavelengths (355, 532, and 1064 nm) on the variation of morphological features and yields of colloids obtained through the ablation of Ag, Au, and Ag_50_Au_50_ targets conducted in a medium of DW and aqueous NaCl solution using a picosecond laser. Our findings demonstrated that the irradiation laser wavelengths of the UV region are more beneficial to produce smaller NPs due to the fragmentation effects. Conversely, the wavelengths lying in the NIR region are the ideal choice to obtain high ablation yield due to the low absorption by previously generated NPs. Furthermore, NPs sizes were smaller at lower irradiation laser wavelengths than those obtained at higher laser wavelengths. The subsequent development of flexible SERS substrates, utilizing the diverse NPs produced, showcased the superior performance of substrates containing NPs generated at the 1064 nm wavelength, characterized by enhanced yield and larger particle size. However, Au NPs in DW demonstrated optimal long-term stability, maintaining their SERS signal integrity for up to 60 days. Further, chemical warfare simulant MS and DMMP detection using a 325 nm Raman laser (UV resonance) demonstrated superior performance.

## Supporting Information

File 1 Additional figures and tables.

## Data Availability

All data that supports the findings of this study is available in the published article and/or the supporting information to this article.
