# Supplementary material for: Effect of wavelength and liquid on formation of Ag, Au, Ag/Au nanoparticles via picosecond laser ablation and SERS-based detection of DMMP
Source: Beilstein J Nanotechnol. 2024 Aug 19;15:1054–69. doi: 10.3762/bjnano.15.86 (PMC11346305; doi:10.3762/bjnano.15.86)
Supplement: File 1 — Additional figures and tables. [file Beilstein_J_Nanotechnol-15-1054-s001.pdf]

BEILSTEIN JOURNAL OF NANOTECHNOLOGY

Supporting Information

for

Effect of wavelength and liquid on formation of Ag, Au, Ag/Au nanoparticles via picosecond laser ablation and SERS-based detection of DMMP

Sree Satya Bharati Moram, Chandu Byram and Venugopal Rao Soma

Beilstein J. Nanotechnol. **2024,** *15,* 1054–1069. doi:10.3762/bjnano.15.86

Additional figures and tables

License and Terms: This is a supporting information file under the terms of the Creative Commons Attribution License (https://creativecommons.org/licenses/by/4.0). Please note that the reuse, redistribution and reproduction in particular requires that the author(s) and source are credited and that individual graphics may be subject to special legal provisions.
The license is subject to the *Beilstein Journal of Nanotechnology* terms and conditions: (https://www.beilstein-journals.org/bjnano/terms)

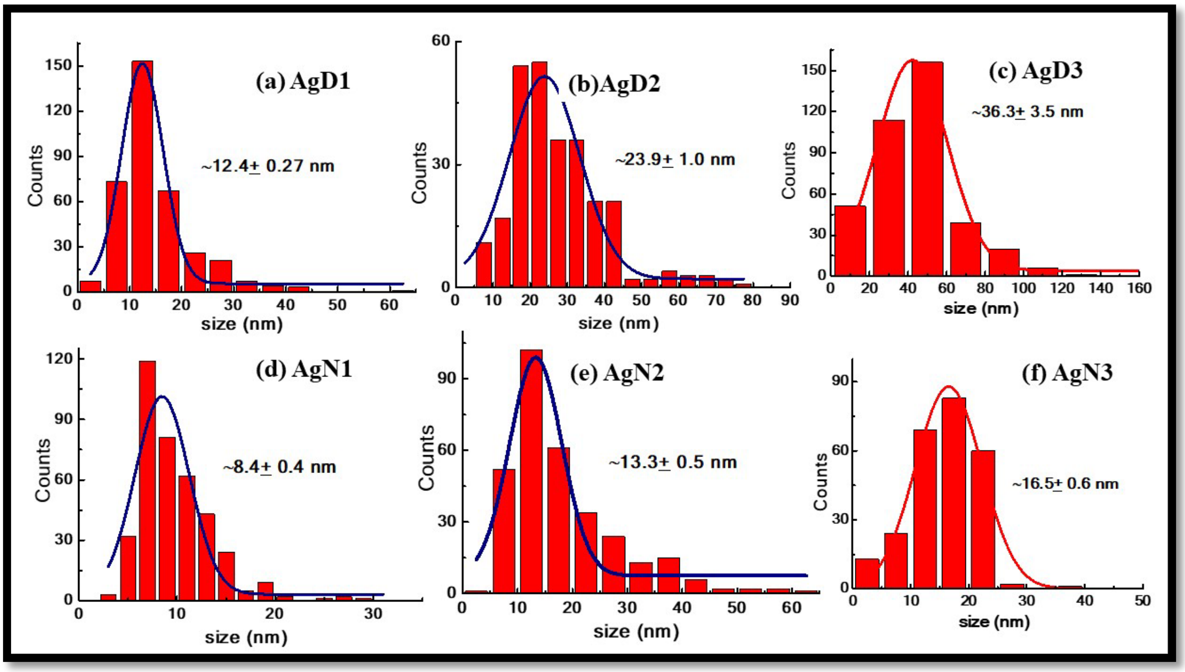

Figure S1: Size distribution histogram plots of Ag NPs [(a) AgD1, (b) AgD2, (c) AgD3, (d) AgN1, (e) AgN2, and (f) AgN3].

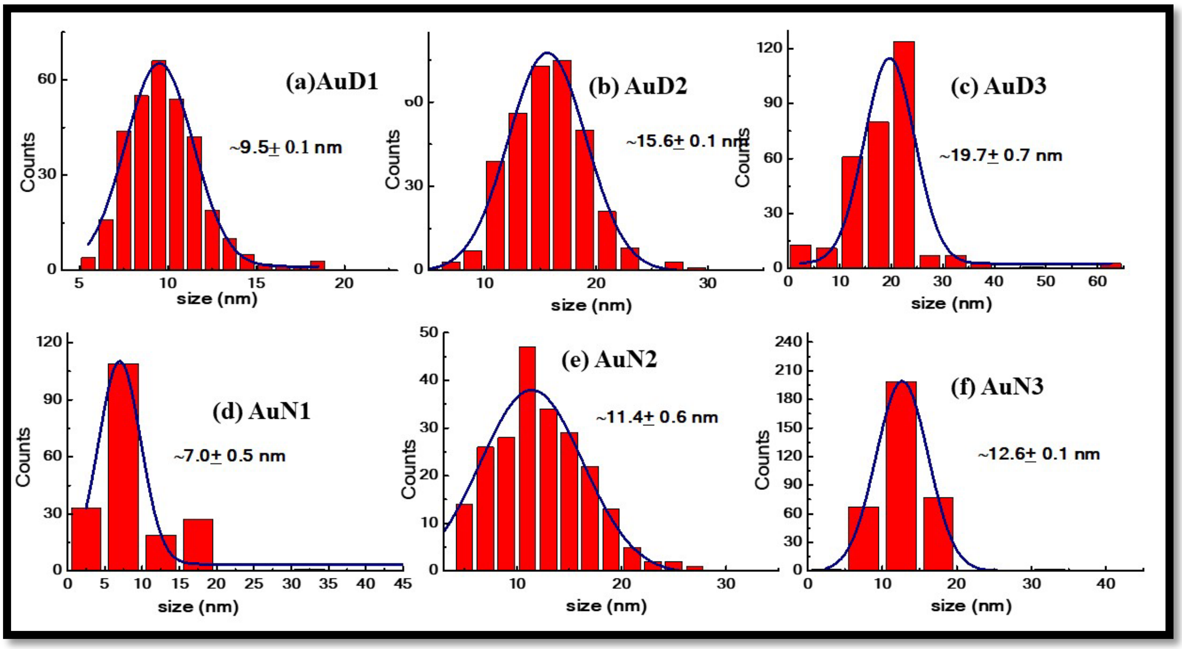

Figure S2: Size distribution histogram plots of Au NPs [(a) AuD1, (b) AuD2, (c) AuD3, (d) AuN1, (e) AuN2, and (f) AugN3].

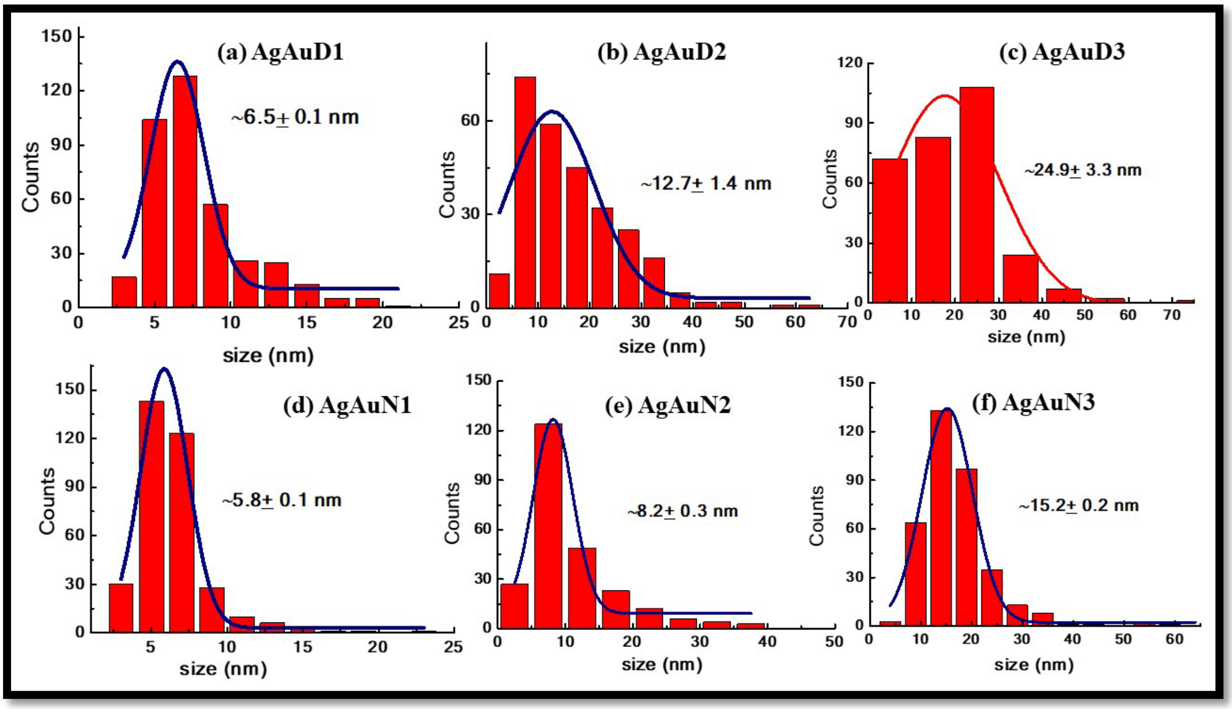

Figure S3: Size distribution histogram plots of AgAu NPs [(a) AgAuD1, (b) AgAuD2, (c) AgAuD3, (d) AgAuN1, (e) AgAuN2, and (f) AgAuN3].

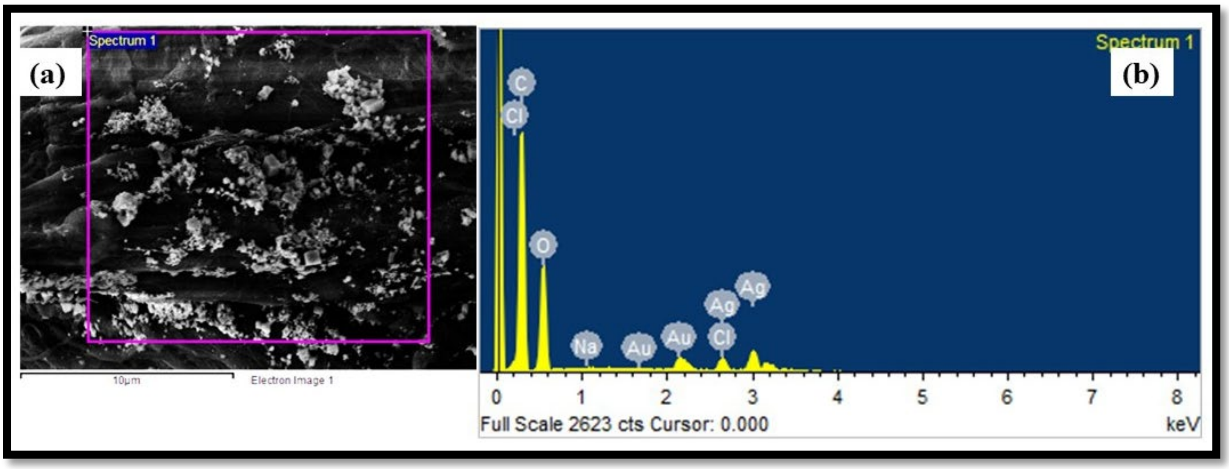

Figure S4: FP with (a) AgAuN3 and the corresponding (b) EDX spectra.

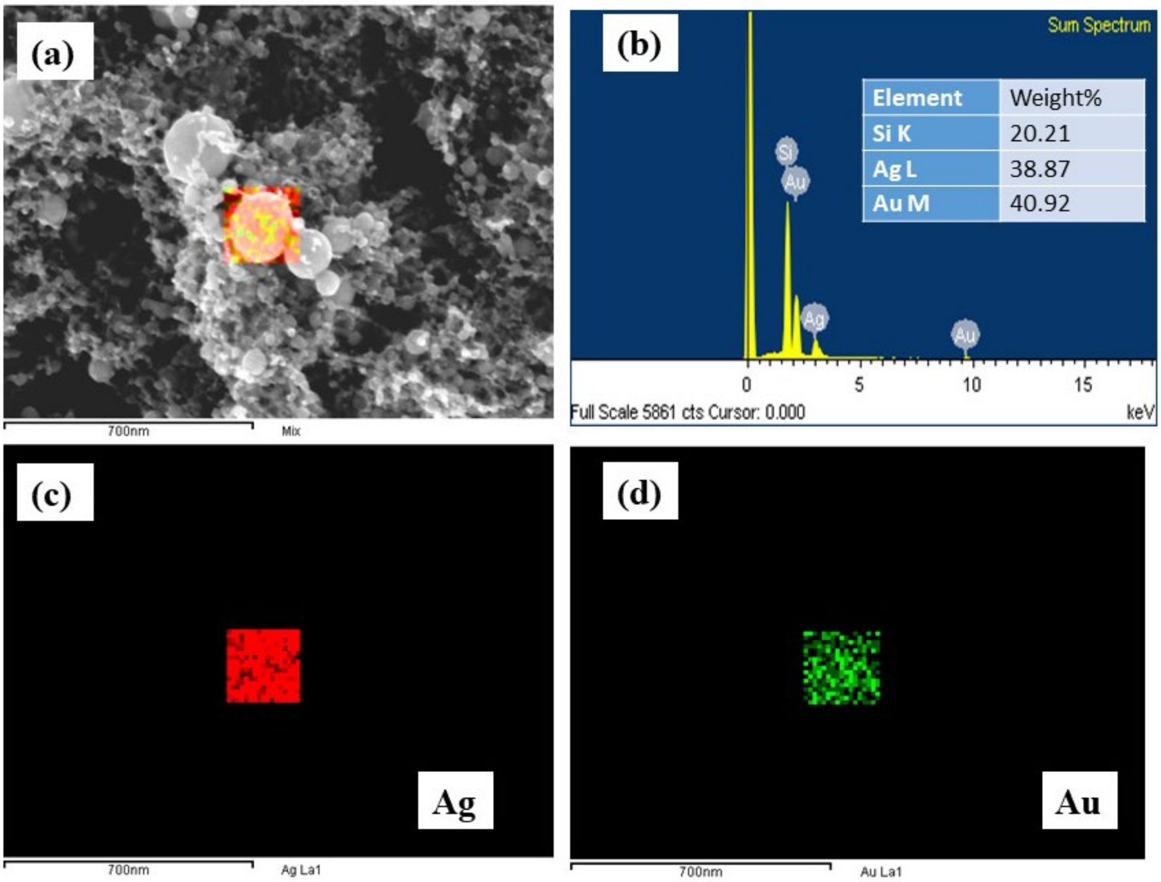

Figure S5: (a) FESEM–DES mapping image of AgAuD3 NPs on SI (b) EDX spectra (inset wt%) (c) Ag (d) Au.

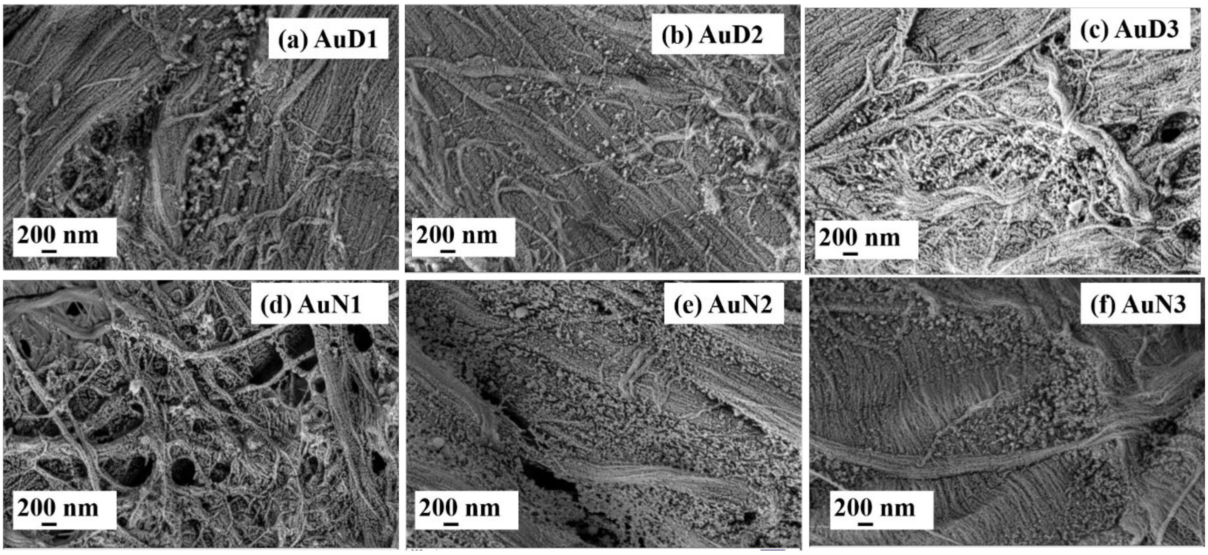

Figure S6: FESEM images of the filter paper loaded with Au NPs obtained in (a)–(c) DW and (d)–(f) NaCl.

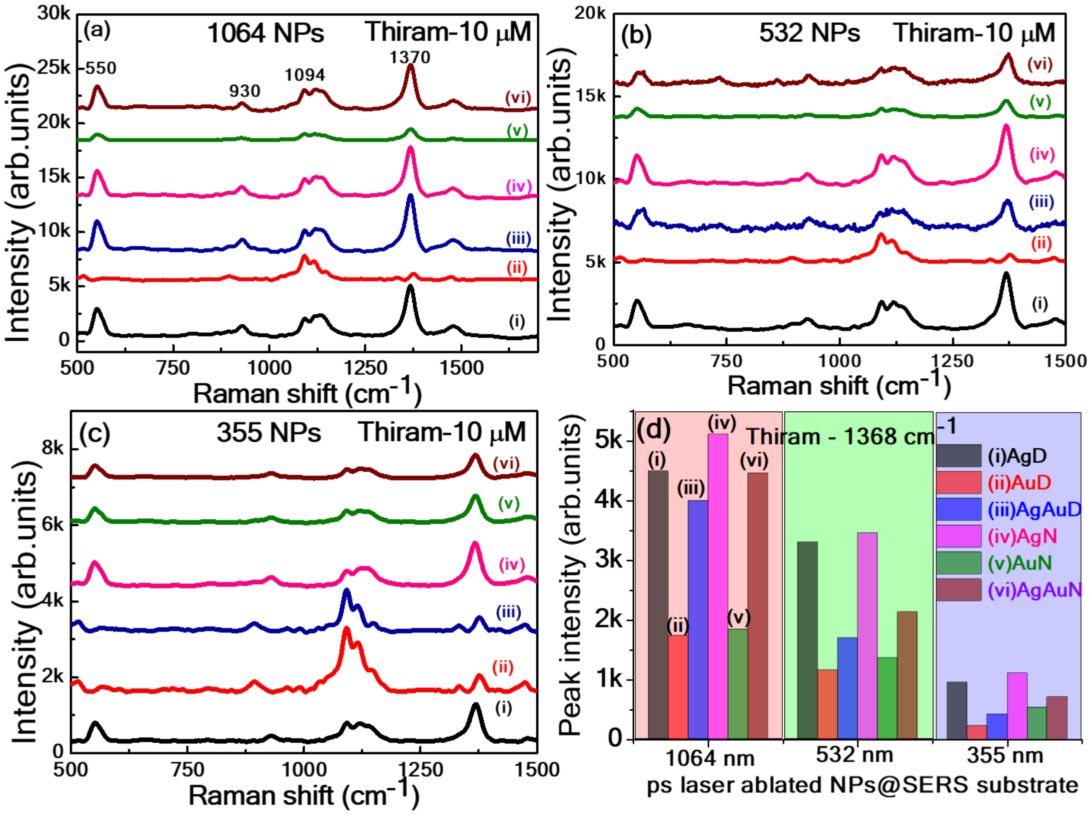

Figure S7: SERS spectra of thiram (10 μM) using filter paper loaded with ps laser-ablated AgD, AuD, AgAuD, AgAuN, AuD, and AuN NPs at (a) 1064, (b) 532, and (c) 355 nm. (d) Prominent peak (1368 cm^{-1}) intensity histogram.

Table S8: MS Raman peak and their assignments [1]

Methyl salicylate (MS): $C_8H_8O_3$

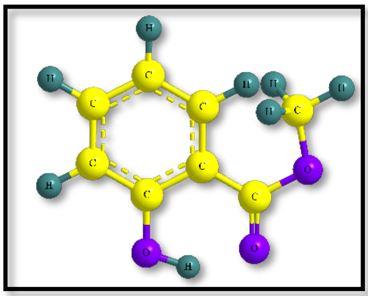

S. No.	Peak position	Assignment
1	562	Out-of-plane of the benzene ring
2	661	Out-of-plane and in-plane of a benzene ring
3	808	Stretching vibration of C–H
4	1033	In-plane of a benzene ring
5	1250	Stretching vibration of C–O

Table S9: DMMP Raman peak and their assignments [2]

Dimethyl methylphosphonate (DMMP): $C_3H_9O_3P$

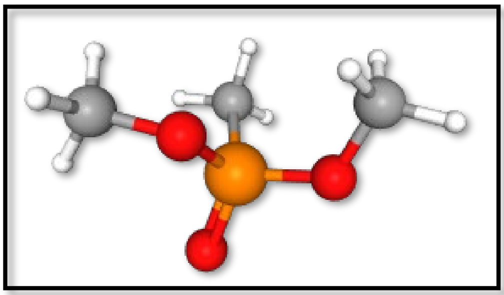

S. No.	Raman Peak position	Assignment
1	714 (strong)	Stretching P–CH_3 + stretching P–O + bending P–O–CH_3
2	794	Asymmetric stretching O–P–O
3	825	Stretching P–CH_3
4	1028	Stretching O–CH3 + asymmetric stretching (O–P–O)
5	1057	Stretching O–CH3 + stretching (O–P–O)

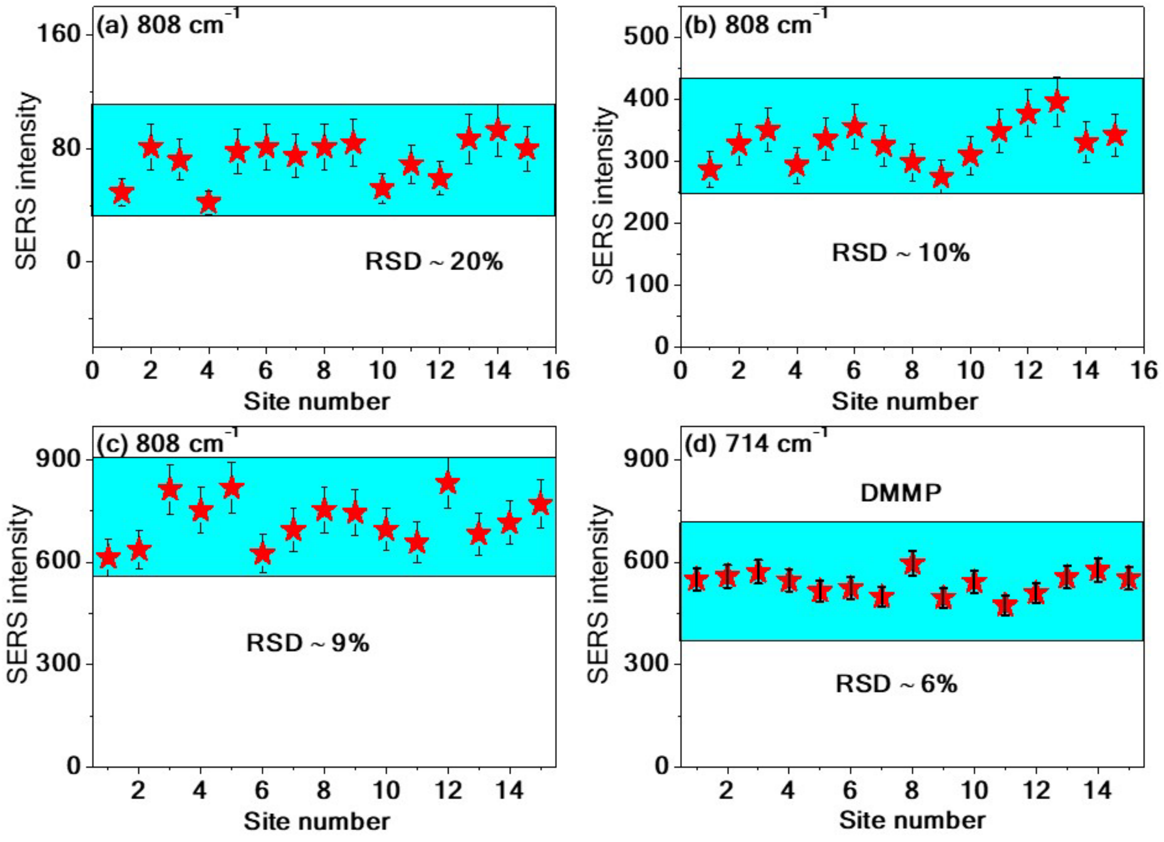

Figure S10: Reproducibility data of SERS MS (1 mM) at the following Raman excitations: (a) 632, (b) 532, and (c) 325 nm. (d) Reproducibility data of SERS DMMP (500 μM) at Raman excitations of 325 nm.

Reference:

(1) Li, Y.; Li, Q.; Wang, Y.; Oh, J.; Jin, S.; Park, Y.; Zhou, T.; Zhao, B.; Ruan, W.; Jung, Y. M. A reagent-assisted method in SERS detection of methyl salicylate. *Spectrochimica Acta Part A: Molecular and Biomolecular Spectroscopy* **2018**, *195*, 172-175. DOI: https://doi.org/10.1016/j.saa.2018.01.073.

(2) Costa, J. C. S.; Ando, R. A.; Sant'Ana, A. C.; Corio, P. Surface-enhanced Raman spectroscopy studies of organophosphorous model molecules and pesticides. *Physical Chemistry Chemical Physics* **2012**, *14* (45), 15645-15651, 10.1039/C2CP42496G. DOI: 10.1039/C2CP42496G.
